# Osteoporosis and stroke: a bidirectional mendelian randomization study

**DOI:** 10.1007/s00774-025-01579-x

**Published:** 2025-01-10

**Authors:** Miao He, Haochuan Yong, Zhidong Cao, Jie Li

**Affiliations:** Department of Orthopedic Surgery, Chongqing Emergency Medical Center (Chongqing University Central Hospital), Jiankang Road 1, Chongqing, 400010 China

**Keywords:** Osteoporosis, Stroke, Genome-wide association study, Mendelian randomization

## Abstract

**Introduction:**

Numerous observational studies have identified a link between osteoporosis and stroke. However, the causal genetic relationship between these conditions remains unclear. This study employs a two-sample bidirectional Mendelian randomization (MR) approach to ascertain the causal relationship between osteoporosis and stroke.

**Materials and Methods:**

We conducted a two-sample Mendelian randomization (MR) study to investigate the potential causal relationship between osteoporosis and stroke, including its subtypes. Genetic data for osteoporosis and stroke, along with their subtypes, were sourced from published genome-wide association studies (GWAS). Single nucleotide polymorphisms (SNPs) demonstrating genome-wide significance (p < 5 × 10^ − 8) and independence (r^2 < 0.001) were selected for further analysis, provided they had an F-statistic ≥ 10. The inverse-variance weighted (IVW) method was employed to evaluate causality, with results reported as odds ratios (ORs). Heterogeneity was assessed using Cochran’s Q test, while pleiotropy was tested using the MR-Egger intercept test. A leave-one-out sensitivity analysis was performed to ensure the robustness of the results.

**Results:**

Employing the IVW method, MR Egger method, and median-weighted method, we found no significant bidirectional causal relationship between osteoporosis and stroke or its subtypes, irrespective of the inclusion of potential pleiotropic SNPs. Sensitivity analyses affirmed the reliability and stability of these findings.

**Conclusion:**

Our study findings indicate that there is no direct causal relationship between osteoporosis and stroke or its subtypes in either direction. Based on our results, although no direct link was found, secondary effects do exist.

**Supplementary Information:**

The online version contains supplementary material available at 10.1007/s00774-025-01579-x.

## Introduction

Osteoporosis and stroke are significant public health concerns worldwide. [1, 2] Stroke is a leading cause of disability and death, affecting approximately 15 million people each year, and its prevalence is expected to increase with the aging population. [3, 4] While men are more likely to experience a stroke, women often face worse outcomes in terms of disability and mortality. [5–7] Osteoporosis, characterized by decreased bone density and increased fracture risk, frequently develops after a stroke, especially on the paralyzed side of the body [8–10]. This form of osteoporosis is more prevalent and severe than osteoporosis that occurs solely due to aging, leading to higher fracture rates, increased mortality, and a reduced quality of life. [11–13]

There is a notable interrelationship between stroke and osteoporosis [14, 15]. Stroke can lead to a reduction in bone mineral density (BMD), increasing the risk of osteoporosis and fractures [16]. Both conditions share common risk factors, such as aging, physical inactivity, and the presence of inflammatory cytokines [17, 18]. This overlap suggests a complex interaction between stroke and osteoporosis, which may impact patient outcomes and complicate management strategies [19].

Understanding the causal relationship between stroke and osteoporosis is crucial for developing effective prevention and management strategies. However, this relationship remains unclear due to various confounding factors that may influence both conditions. To address this uncertainty, Mendelian randomization (MR) has been proposed as a method to clarify causality [20]. MR utilizes genetic variants to determine the direction and strength of causal relationships, thereby minimizing the impact of confounders that typically affect observational studies [21, 22].

This study aims to explore the potential bidirectional causal effects between osteoporosis (as measured by BMD), and different types of stroke, including ischemic stroke(IS), large vessel stroke (LV-IS), small vessel stroke (SV-IS), cardioembolic stroke (CE-IS), and intracerebral hemorrhage (ICH). Utilizing a two-sample bidirectional Mendelian randomization approach with data from genome-wide association studies (GWAS), the research seeks to clarify these relationships.

The findings may enhance our understanding of the interplay between stroke and osteoporosis, ultimately informing better management and preventive strategies for at-risk patients, thereby improving patient care and outcomes.

## Materials and methods

### Study design and data sources

**Fig. 1:**
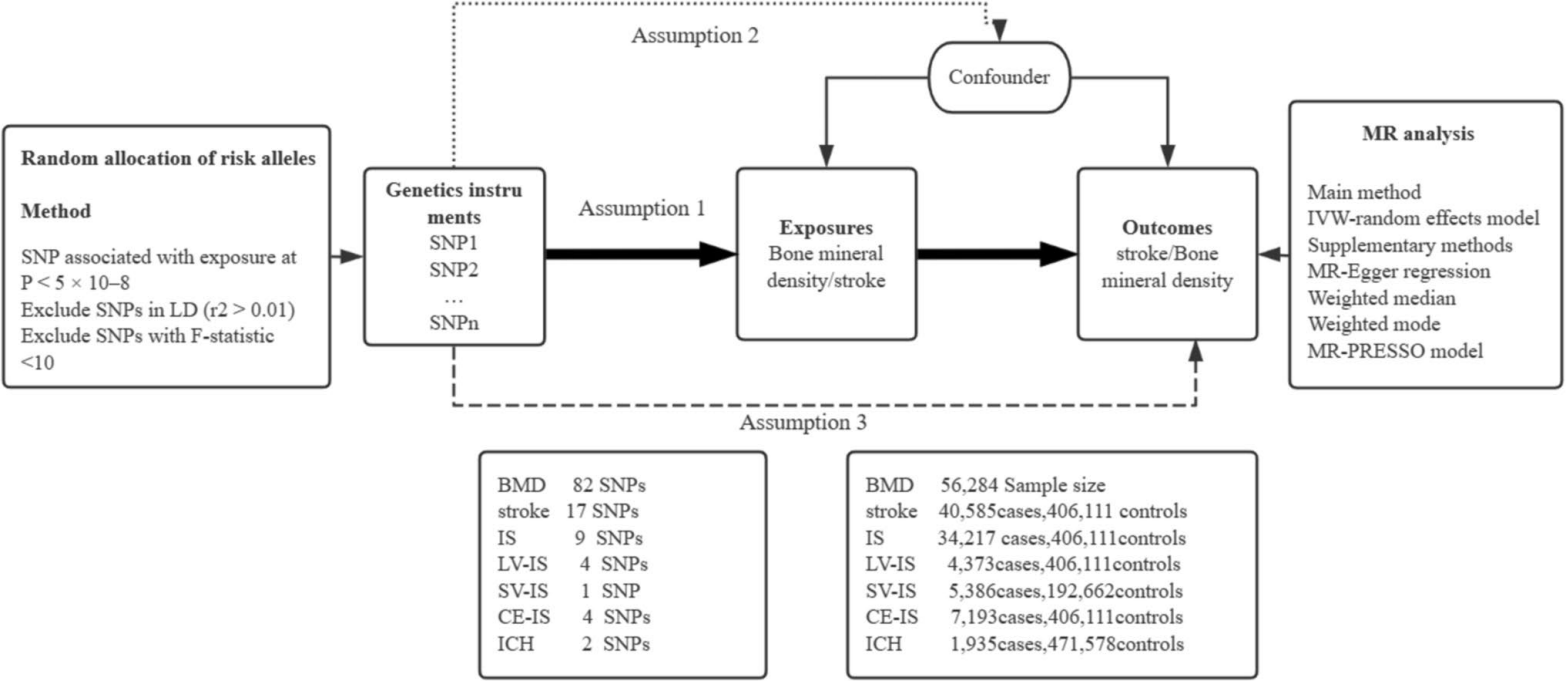
Study Design Overview.Assumption 1: Genetic variants (instrumental variables) must have a strong association with the risk factor being studied.Assumption 2: Genetic variants should not be related to any confounding factors.Assumption 3: Genetic variants should influence the outcome only through the risk factor, not via other pathways.· Note. MR, Mendelian randomization;BMD,Bone mineral density IS, Ischemic stroke; LV-IS, Large vessel ischemic stroke; SV-IS, Small vessel ischemic stroke; CE-IS, Cardioembolic ischemic stroke; ICH, Intracerebral hemorrhage; PRESSO, Pleiotropy Residual Sum and Outlier

In this two-sample MR study, we used single nucleotide polymorphisms (SNPs) as instrumental variables (IVs) and GWAS data to determine the causal relationship between osteoporosis and stroke. The study design and assumptions for the MR study are outlined in Fig. 1. The genetic data related to osteoporosis and stroke were obtained from published GWAS studies, with detailed data presented in Table 1.As this study was based on previously published GWAS summary data, approval from an institutional review board was not necessary, and all participants provided informed consent beforehand.

**Table 1:** Data sources used in this study

Exposures or outcome	Sample size (total or cases/controls)	Ancestry	Consortia	PubMed ID or URL of original research	PubMed ID or URL
Total body bone mineral density	56,284	European	GWAS meta-analysis	29,304,378	https://gwas.mrcieu.ac.uk/datasets/ebi-a-GCST005348/
stroke	446,696	European	GWAS meta-analysis	29,531,354	https://gwas.mrcieu.ac.uk/datasets/ebi-a-GCST005838/
Ischemic stroke	440,328	European	GWAS meta-analysis	29,531,354	https://gwas.mrcieu.ac.uk/datasets/ebi-a-GCST006908/
Large vessel ischemic stroke	150,765	European	GWAS meta-analysis	29,531,354	https://gwas.mrcieu.ac.uk/datasets/ebi-a-GCST005840/
Small vessel ischemic stroke	198,048	European	GWAS meta-analysis	29,531,354	https://gwas.mrcieu.ac.uk/datasets/ebi-a-GCST005841/
Cardioembolic ischemic stroke	211,763	European	GWAS meta-analysis	29,531,354	https://gwas.mrcieu.ac.uk/datasets/ebi-a-GCST005842/
Intracerebral hemorrhage	473,513	European	GWAS meta-analysis	34,594,039	https://gwas.mrcieu.ac.uk/datasets/ebi-a-GCST90018870/

### Genetic instrument selection

A bidirectional two-sample MR study was conducted to investigate the causal role of Osteoporosis in stroke. Figure 1 provides an overview of the bidirectional MR design. MR analysis is predicated on the following three essential assumptions. First, the genetic variants selected as instrumental variables must be associated with the relevant exposure. Second, the single nucleotide polymorphisms (SNPs) of genetic variants should not be associated with unmeasured confounders between exposure and outcome. Lastly, genetic variations influence outcomes exclusively through the exposure, rather than through other pathways.We applied a genome-wide significance threshold of P < 5 × 10^–8^, ensuring robustness against weak instrument bias, and restricted the selection to genetic variants with a minor allele frequency (MAF) > 0.01 to identify instrumental variables for osteoporosis and stroke [24].

*Linkage Disequilibrium (LD) Control*: To control for linkage disequilibrium, a clumping procedure was implemented. This process involved selecting SNPs within a 10,000 kilobase (kb) window and retaining those with the lowest p-values among pairs of SNPs in high LD (R^2 < 0.001) [25]. The data for this procedure was drawn from individuals of European ancestry to maintain consistency in the genetic background.

*Proxy SNPs*: In cases where target SNPs were missing in the outcome genome-wide association studies (GWAS), proxy SNPs were utilized. These proxy SNPs were chosen based on their strong linkage disequilibrium (R^2 > 0.8) with the target SNPs, ensuring that the genetic variation being studied was accurately represented even in the absence of the original SNP [26].

*Harmonization*: Harmonization of the SNPs was performed to ensure that the effect alleles were consistent across both the exposure and outcome datasets. This step was crucial for maintaining the validity of the genetic associations being studied. SNPs that were discordant or palindromic, where the alleles could not be reliably aligned, were excluded from the analysis to prevent potential errors in interpretation.

*F-statistic Calculation*: To assess the reliability and strength of the genetic instruments, F-statistics were calculated for each SNP. SNPs with F-statistics < 10 were excluded from further analysis, as such weak instruments could introduce bias and reduce the robustness of the results [27].

### MR analysis

In this Mendelian Randomization (MR) analysis, a bidirectional approach was employed to explore the causal relationship between Stroke and bone mineral density (BMD). By analyzing both directions, the study aimed to determine whether stroke influences BMD and vice versa, providing a comprehensive understanding of their interaction.

The primary method utilized was the random-effects inverse variance weighted (IVW) technique. This method was chosen for its effectiveness in estimating causal effects, especially when there is heterogeneity among the genetic instruments.[24]The random-effects model allows for variability across SNPs, ensuring more robust causal estimates.

To further enhance the reliability of the findings, additional MR methods were applied. MR-Egger regression was used to address directional pleiotropy, as it is less susceptible to biases arising from genetic variants that influence the outcome through other pathways [25]. Median-based estimators, including the weighted median and mode, were also employed for their robustness against outliers, providing a safeguard against extreme values that could skew the results.

To correct for potential pleiotropic effects, the MR-PRESSO (Pleiotropy RESidual Sum and Outlier) outlier test was implemented. This test detects and adjusts for horizontal pleiotropy, where genetic variants affect the outcome through pathways unrelated to the exposure of interest. By applying MR-PRESSO, the accuracy of the causal estimates was improved, ensuring that the results were not confounded by pleiotropic effects [26].

### Heterogeneity, pleiotropy, and sensitivity analysis

To assess horizontal pleiotropy in the Mendelian Randomization (MR) analysis, MR-Egger regression and MR-PRESSO global tests were employed. The intercept from MR-Egger regression served as an indicator of the average pleiotropic effect across SNPs, while a skewed funnel plot provided visual evidence of potential pleiotropy [27]. These methods collectively ensured that the genetic instruments' influence on the outcome was not through alternative pathways unrelated to the exposure.

For heterogeneity detection, Cochrane’s Q statistic was used to evaluate variability across the genetic instruments. A leave-one-out analysis was conducted to identify whether any single SNP disproportionately influenced the observed association. This approach helped maintain the robustness of the causal inference by ensuring that the results were not driven by individual outliers [28].

Confounder Exclusion: To minimize bias, SNPs associated with potential confounders or related outcomes, such as coronary artery disease, hypertension, and diabetes, were excluded based on data from the GWAS Catalog website (https://www.ebi.ac.uk/gwas). (Supplementary Tables 1, 2) This step helps ensure that the genetic instruments used are not influenced by factors that could confound the results.

### Statistical significance

All statistical analyses were performed using the TwoSampleMR package in R version 4.4.0 (R Foundation for Statistical Computing).A p-value < 0.05 was considered statistically significant.

## Results

### Effect of bone mineral density on stroke

**Fig. 2:**
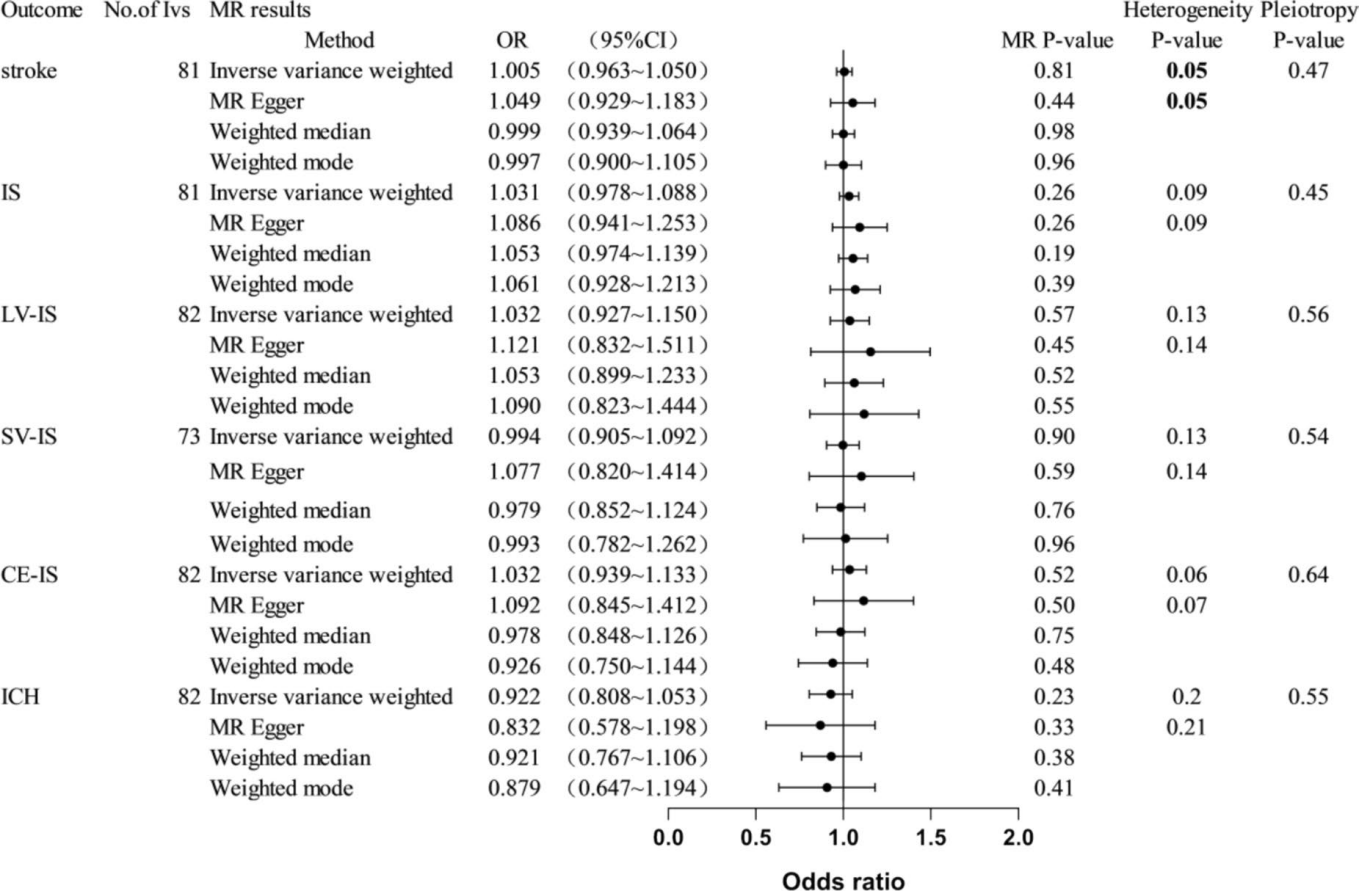
MR estimates from each method of assessing the causal effects of Bone Mineral Density on stroke and subtypes. Note. OR, Odds ratios; CI, confidence interval

In our study, after taking into account the independence and LD of genetic variants,we used 82 independent SNPs as instrumental variables (IVs) for bone mineral density (BMD) (Table 2, Fig. 2). The MR analysis results indicated no significant relationship between genetically predicted BMD and overall stroke or its subtypes. The primary IVW results showed no significant association between BMD and stroke [IVW odds ratio (OR): 1.005, 95% confidence interval (CI): 0.963 ~ 1.050, P = 0.81], BMD and IS (OR: 1.031, 95% CI: 0.978 ~ 1.088, P = 0.26), LV-IS (OR: 1.032, 95% CI: 0.927 ~ 1.150, P = 0.57), SV-IS (OR: 0.994, 95% CI: 0.905 ~ 1.092, P = 0.90), CE-IS (OR: 1.032, 95% CI: 0.939 ~ 1.133, P = 0.52), and ICH (OR: 0.922, 95% CI: 0.808 ~ 1.053, P = 0.23). Similar results were observed with MR-Egger regression and median-based estimators (weighted median and weighted mode) (Fig. 2). The horizontal pleiotropy test results indicated no directional pleiotropy (Fig. 2).

**Table 2:** MR Heterogeneity and Pleiotropy Analysis of the Relationship Between Bone Mineral Density and Stroke

Exposure	Outcome	No.of Ivs	Heterogeneity tests		Directional horizontal pleiotropy test
			Methods	Cochran’sQ (P)	MR-Egger intercept (P)
TB-BMD	Stroke	81	MR Egger	**100.35(0.05)**	− 2.44E-03(0.47)
			IVW	**101.04(0.05)**	
TB-BMD	IS	81	MR Egger	96.54(0.09)	− 3.02E-03(0.45)
			IVW	97.26(0.09)	
TB-BMD	LV-IS	82	MR Egger	94.16(0.13)	− 4.78E-03(0.56)
			IVW	94.56(0.14)	
TB-BMD	SV-IS	73	MR Egger	84.64(0.13)	− 4.55E-03(0.54)
			IVW	85.10(0.14)	
TB-BMD	CE-IS	82	MR Egger	100.56(0.06)	− 3.29E-03(0.64)
			IVW	100.84(0.07)	
TB-BMD	ICH	82	MR Egger	90.56(0.20)	5.99E-03(0.55)
			IVW	90.96(0.21)	
Stroke	TB-BMD	17	MR Egger	**37.16(1.20E-03)**	4.64E-04(0.98)
			IVW	**37.16(1.99E-03)**	
IS	TB-BMD	9	MR Egger	**15.95(0.03)**	2,16E-02(0.40)
			IVW	**17.81(0.02)**	
LV-IS	TB-BMD	4	MR Egger	2.61(0.27)	4.53E-02(0.26)
			IVW	5.81(0.12)	
SV-IS	TB-BMD	1	MR Egger	NA	NA
			IVW	NA	
CE-IS	TB-BMD	4	MR Egger	3.07(0.22)	− 5.21E-04(0.96)
			IVW	3.07(0.38)	
ICH	TB-BMD	2	IVW	**5.89(0.02)**	NA

TB-BMD,Total body bone mineral density;IVW, inverse-variance-weighted

The bold part is to emphasize “The P-value of Cochran’s Q (P) < 0.05”, indicates the presence of heterogeneity. We then used MR-PRESSO to remove outliers and re-conducted MR analysis to substantiate the conclusions.”

In Cochran’s Q heterogeneity test, heterogeneity was detected in the BMD-overall stroke association (IVW = 101.04, P = 0.05; MR-Egger = 100.35, P = 0.05, Fig. 2, Table 2). Therefore, we conducted an MR-PRESSO test to further examine this relationship. The MR-PRESSO results indicated no significant outliers (Global Test P value = 0.0606). No outliers were identified, so the results for the outlier-corrected MR are set to NA. The leave-one-out sensitivity analysis results showed that no single SNP had a potential impact on the final results. Supplementary Fig. 1 describes the scatter plots, funnel plots, and leave-one-out analysis plots of BMD on overall stroke and stroke subtypes.

To further strengthen our MR assumption, we examined the traits related to our instrumental SNPs. Traits association analysis (see Supplementary Table 1) showed that rs2252865,rs2873195 in TB-BMD, are associated with psychiatric diseases and hyperlipemia, which may have some effect on the risk of overall stroke or its subtypes. Sensitivity analysis by removing the SNPs revealed similar results.(see Supplementary Fig. 3).

### Effect of stroke on bone mineral density

**Fig. 3:**
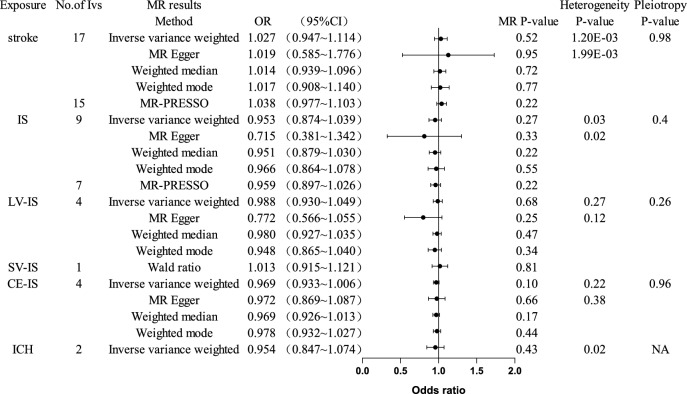
MR estimates from each method of assessing the causal effects of stroke and subtypes on Bone Mineral Density. Note.OR, Odds ratios; CI, confidence interval

After taking into account the independence and LD of genetic variants,we obtained 17, 9, 4, 1, 4, and 2 SNPs from GWAS to serve as IVs for overall stroke, IS, LV-IS, SV-IS, CE-IS, and ICH, respectively (see Fig. 3, Table 2). The MR analysis results indicated no significant relationship between genetically predicted overall stroke and its subtypes with bone mineral density (BMD). The primary IVW results showed no significant association between stroke and BMD (OR: 1.027, 95% CI: 0.947 ~ 1.114, P = 0.52), IS and BMD (OR: 0.953, 95% CI: 0.874 ~ 1.039, P = 0.27), LV-IS and BMD (OR: 0.988, 95% CI: 0.930 ~ 1.049, P = 0.68), SV-IS and BMD (OR: 1.013, 95% CI: 0.915 ~ 1.121, P = 0.81), CE-IS and BMD (OR: 0.969, 95% CI: 0.933 ~ 1.006, P = 0.10), and ICH and BMD (OR: 0.954, 95% CI: 0.847 ~ 1.074, P = 0.43). Similar results were observed with MR-Egger regression and median-based estimators (weighted median and weighted mode) (Fig. 3, Table 2). All Egger regression tests were negative (p > 0.05; Fig. 3), indicating that our MR results were not influenced by horizontal pleiotropy.

In the Cochran’s Q heterogeneity test, heterogeneity was detected in overall stroke, IS, and ICH. For stroke (IVW = 37.16, P = 1.99E-03; MR-Egger = 37.16, P = 1.20E-03), IS (IVW = 17.81, P = 0.02; MR-Egger = 15.95, P = 0.03), and ICH (IVW = 5.89, P = 0.02) (Fig. 3, Table 2). Therefore, we conducted an MR-PRESSO test to further examine these relationships. For overall stroke, SNPs rs3184504 and rs42039 were excluded, and for IS, SNPs rs3184504 and rs4942561 were excluded. After removing the outlier variants, we still found no causal relationship between stroke and reduced BMD (see Fig. 3, Table 2). The leave-one-out sensitivity analysis results showed that no single SNP had a potential impact on the final results. Supplementary Fig. 2 describes the scatter plots, funnel plots, and leave-one-out analysis plots of stroke and its subtypes on BMD.

To further strengthen our MR assumption, we examined the traits related to our instrumental SNPs. The results of the trait association analysis (see Supplementary Table 2) indicated that no SNPs,which may have some impact on OP.

## Discussion

This study used bidirectional Mendelian randomization to examine the causal relationship between osteoporosis and the risk of stroke, ischemic stroke (IS), large vessel ischemic stroke (LV-IS), small vessel ischemic stroke (SV-IS), cardioembolic ischemic stroke (CE-IS), and intracerebral hemorrhage (ICH) in either direction. Analysis of summary data from GWASs revealed that genetic changes in bone mineral density were not causally associated with an increased risk of stroke or its subtypes in a bidirectional manner. Sensitivity analyses confirmed the reliability of these results.

The relationship between stroke and osteoporosis has long been a topic of interest in the medical community. Although our study found no genetic causal relationship between stroke and osteoporosis, previous observational studies have shown a strong correlation between the two conditions [14, 15]. Based on previous research, we believe that this clinically observed association may be a secondary effect of stroke rather than an intrinsic direct causal relationship.

First, decreased physical activity is one of the major contributors to post-stroke osteoporosis [29]. Evidence over the past few decades has shown a significant reduction in bone mineral density in stroke survivors, particularly in the limbs on the paralyzed side [30, 31]. At the same time, some studies have found that bone density in the non-paralyzed side does not decrease, and may even increase [32, 33]. Yamada et al. reported increased lumbar spine bone density in 100 stroke patients [34]. A meta-analysis showed that bone mineral density in the distal radius of stroke patients' non-paralyzed side did not decrease [35]. This is because the decreased mobility and mechanical loading of the paralyzed side promote bone resorption and inhibit bone formation, leading to rapid bone loss [36]. Meanwhile, the non-paralyzed side compensates for more physical activity and mechanical load in daily life, leading to an increase in bone density [36]. These findings support our study's results, suggesting that stroke impacts osteoporosis through secondary effects rather than a direct causal relationship.

Another major factor by which stroke influences osteoporosis is calcium homeostasis, particularly vitamin D deficiency [37]. Stroke patients have limited sun exposure as they are more likely to remain indoors [38]. A study by Poole et al. found that most acute stroke patients had vitamin D deficiency [39]. This is also a secondary consequence of stroke, rather than a direct impact of stroke on osteoporosis.

Another factor by which stroke affects osteoporosis is calcium homeostasis, particularly vitamin D deficiency [37]. Stroke patients have limited sun exposure due to their increased likelihood of staying indoors [38]. A study by Poole et al. found that most acute stroke patients suffer from vitamin D deficiency [39]. This, too, is a secondary consequence of stroke, rather than a direct effect of stroke on osteoporosis.

There is also research suggesting that inflammation plays a role in the development of osteoporosis in stroke patients, the inflammatory hypothesis. Stroke can trigger the production of inflammatory markers such as C-reactive protein (CRP), interleukin-6 (IL-6), and tumor necrosis factor-alpha (TNF-α), which stimulate osteoclastogenesis and inhibit osteoblast function, leading to accelerated bone resorption and osteoporosis [40–42]. However, a Mendelian randomization study by Huang et al. showed no causal relationship between high-sensitive CRP and low bone mineral density. Our study also indicates that there is no inherent relationship between stroke and osteoporosis. Therefore, whether inflammation is an intrinsic mechanism by which stroke leads to osteoporosis remains debated, and further research is needed to explore the potential inflammatory mechanisms underlying osteoporosis development in stroke patients.

To our knowledge, this is the first two-sample MR study to analyze the bidirectional causal relationship between osteoporosis and stroke.Our study has several strengths. First,The MR method avoids limitations of traditional observational research, such as residual confounding and reverse causality. Second,The use of large GWAS datasets for osteoporosis and stroke provides more precise effect size estimates compared to smaller or individual-level data studies.However, there are some limitations of our study. Firstly, Choosing SNPs from different large-sample GWAS studies may increase the risk of sample overlap between exposure and outcome variables, which may bias the results. Secondly, the study was unable to assess the causal effect of intracerebral hemorrhage (ICH) on osteoporosis due to the lack of suitable single nucleotide polymorphisms (SNPs) available for analysis. Lastly, the study population was exclusively of European descent, which limits the generalizability of the findings to other ethnic groups. Future research should aim to include more diverse populations and explore additional genetic variants to provide a more comprehensive understanding.

In conclusion, our study findings indicate that there is no direct causal relationship between osteoporosis and stroke or its subtypes in either direction. Based on our results, although no direct causal link was found, secondary causal effects do exist. These secondary effects are related to reduced motor function and mechanical load on the paralyzed limbs following stroke, as well as decreased exposure to sunlight, all of which contribute to the relationship between osteoporosis and stroke.

## Supplementary Information

Below is the link to the electronic supplementary material.

Supplementary file1 (DOCX 2001 KB)

## Data Availability

Publicly available datasets were analyzed in this study. This data can be found at: All GWAS summary statistics can be downloaded from open GWAS for exposures (https://gwas.mrcieu.ac.uk/), GWAS catalog (https://www.ebi.ac.uk/gwas/ and https://data.bris.ac.uk/).
