# Supplementary material for: Osteoporosis and stroke: a bidirectional mendelian randomization study
Source: J Bone Miner Metab. 2025 Jan 10;43(3):256–64. doi: 10.1007/s00774-025-01579-x (PMC12089162; doi:10.1007/s00774-025-01579-x)
Supplement: Supplementary file 1 — Supplementary file1 (DOCX 2001 KB) [file 774_2025_1579_MOESM1_ESM.docx]

**Supplementary Figure 1** depicts scatter plots, funnel plots, and leave-one-out analyses of Bone Mineral Density on Stroke and subtypes.


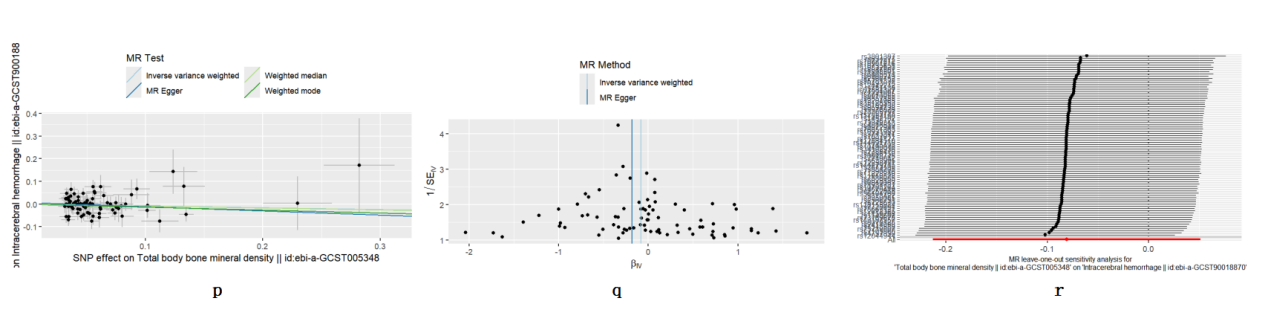

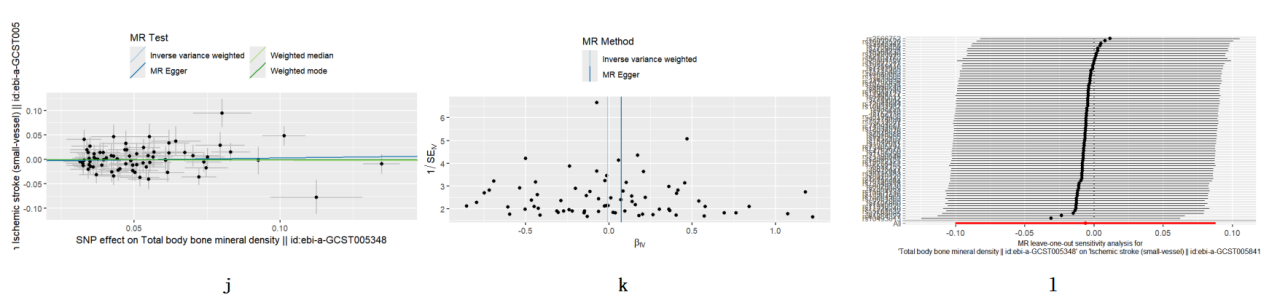

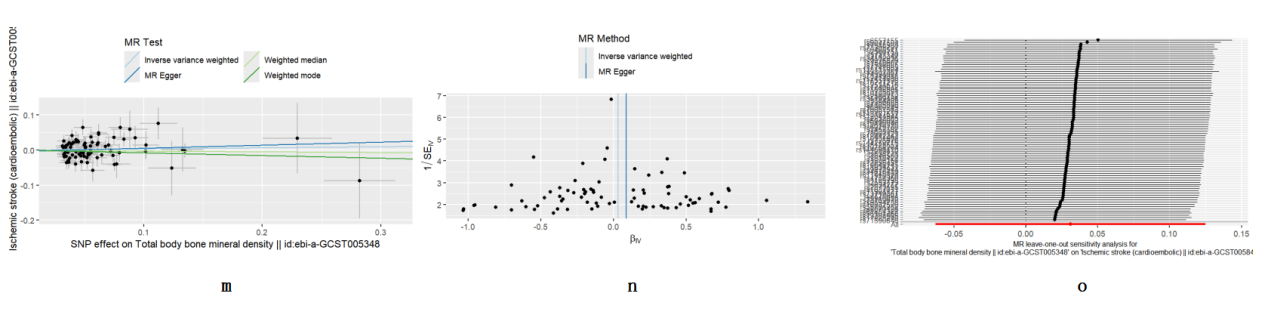

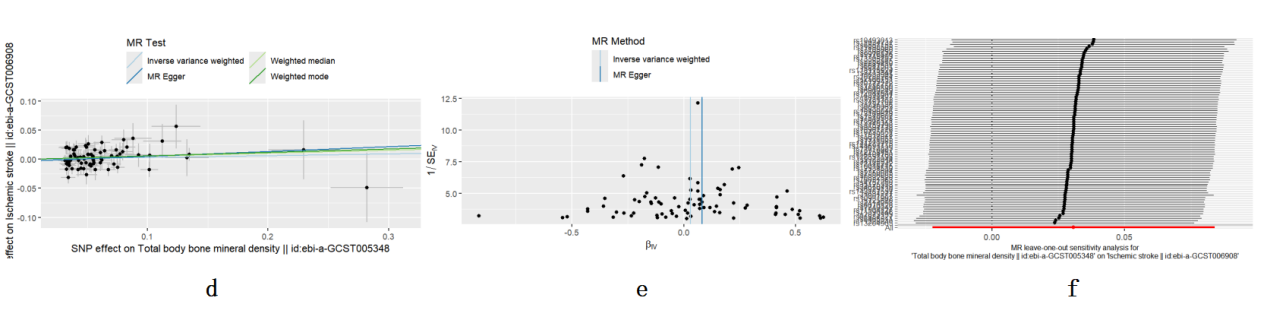

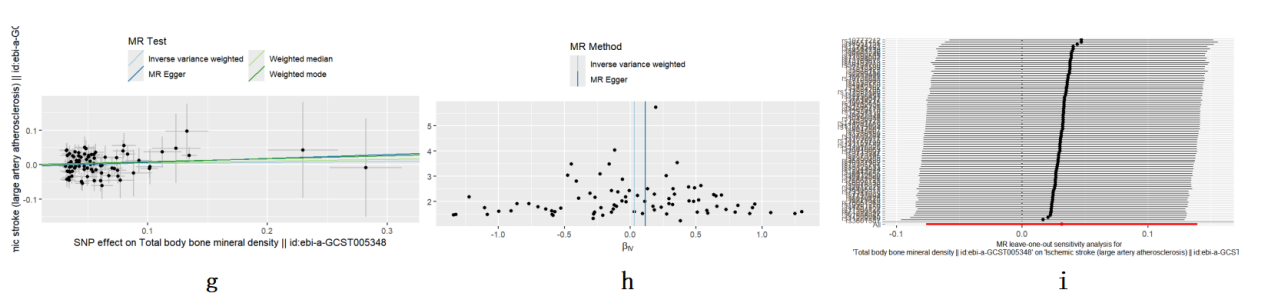

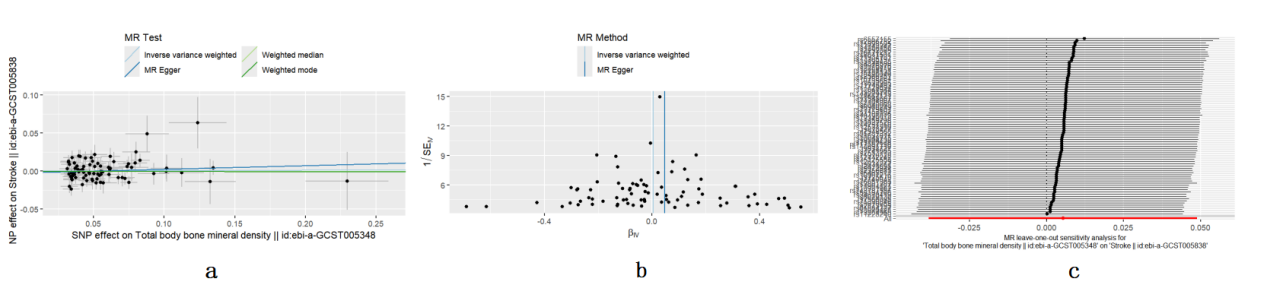


TB-BMD on Stroke:（a）scatter plots, (b) funnel plots, (c) leave-one-out plots

TB-BMD on IS:（d）scatter plots, (e) funnel plots, (f) leave-one-out plots

TB-BMD on LV-IS:（g）scatter plots, (h) funnel plots, (i) leave-one-out plots

TB-BMD on SV-IS:（j）scatter plots, (k) funnel plots, (l) leave-one-out plots

TB-BMD on CE-IS:（m）scatter plots, (n) funnel plots, (o) leave-one-out plots

TB-BMD on ICH:（p）scatter plots, (q) funnel plots, (r) leave-one-out plots

**Supplementary Figure 2** depicts scatter plots, funnel plots, and leave-one-out analyses of Stroke and subtypes on Bone Mineral Density.


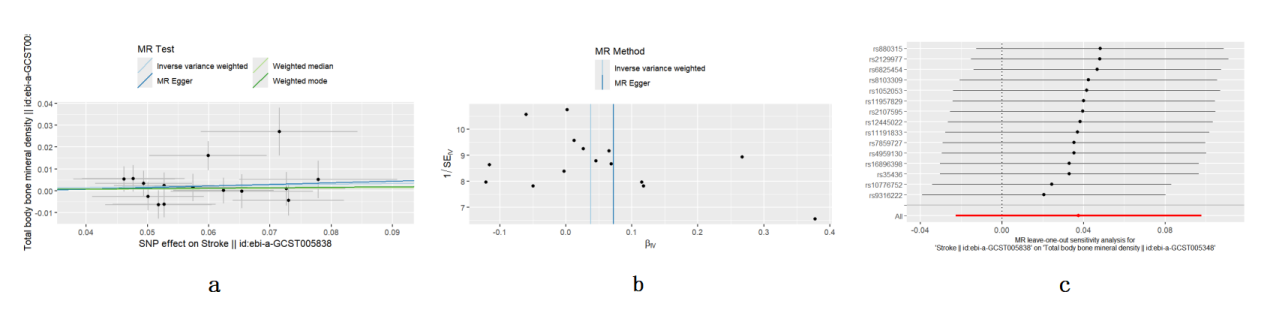

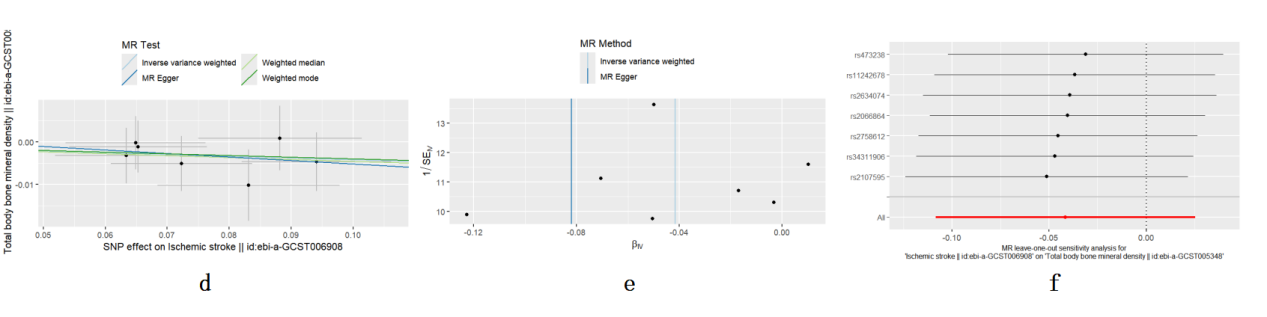

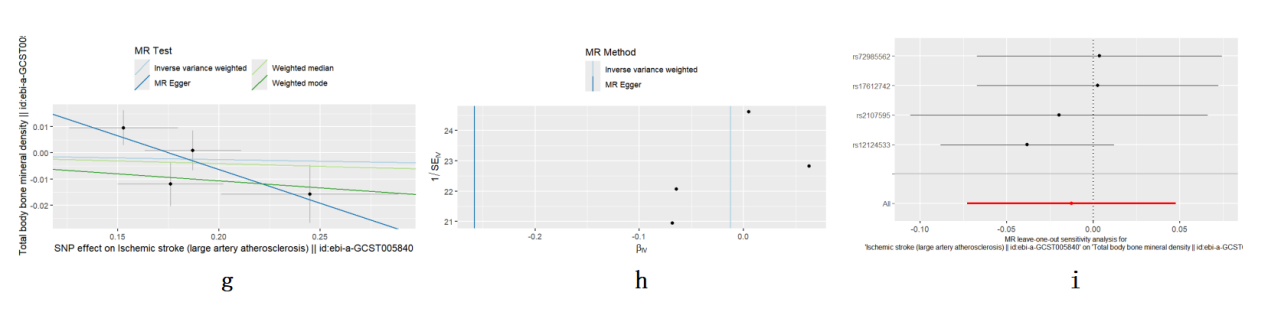

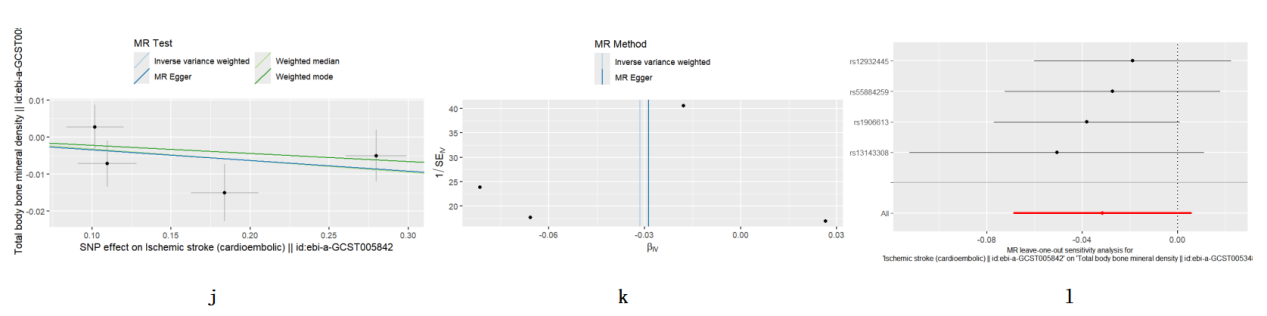


Stroke on TB-BMD:（a）scatter plots, (b) funnel plots, (c) leave-one-out plots

IS on TB-BMD:（d）scatter plots, (e) funnel plots, (f) leave-one-out plots

LV-IS on TB-BMD:（g）scatter plots, (h) funnel plots, (i) leave-one-out plots

CE-IS on TB-BMD:（j）scatter plots, (k) funnel plots, (l) leave-one-out plots

**Supplementary Figure 3.**

MR estimates from each method of assessing the causal effects of Bone Mineral Density on stroke and subtypes after removal of potentially pleiotropic SNPs.

MR estimates from each method of assessing the causal effects of Bone Mineral Density on stroke and subtypes. MR, Mendelian randomization; IS, Ischemic stroke; LV-IS, Large vessel ischemic stroke; SV-IS, Small vessel ischemic stroke; CE-IS, Cardioembolic ischemic stroke; ICH, Intracerebral hemorrhage; OR, Odds ratios; CI, confidence interval

**Supplementary Table 1.**

TB-BMD SNPs from the GWAS used as genetic instruments in the MR study,and the reported traits of selected instrumental variables of TB-BMD searched in GWAS Catalog.

| SNP | Chr | Pos | Gene | EA | OA | EAF | Beta | Se | P | R2 | F | Trait | Excluded from MR analysis |
| --- | --- | --- | --- | --- | --- | --- | --- | --- | --- | --- | --- | --- | --- |
| rs10493013 | 1 | 22703035 | ZBTB40 | C | T | 0.1811 | 0.1013 | 0.0074 | 4.07E-43 | 0.003318386 | 187.3872418 | bone density | no |
| rs12044944 | 1 | 240581653 | FMN2 | T | C | 0.1916 | 0.0553 | 0.0074 | 7.54E-14 | 0.000991223 | 55.84334064 | bone density | no |
| rs2252865 | 1 | 8422676 | RERE | C | T | 0.6759 | 0.0328 | 0.006 | 4.72E-08 | 0.000530676 | 29.88338253 | attention deficit hyperactivity disorder, unipolar depression, bipolar disorder, autism spectrum disorder, schizophrenia | yes |
| rs2566751 | 1 | 68664913 | WLS | A | T | 0.8727 | -0.0567 | 0.01 | 1.32E-08 | 0.000570865 | 32.14775762 | body height | no |
| rs2566752 | 1 | 68656697 | WLS | C | T | 0.3897 | 0.0721 | 0.0059 | 1.88E-34 | 0.002646249 | 149.3313783 | bone density | no |
| rs4846580 | 1 | 219897941 | SLC30A10 | A | G | 0.5329 | 0.0345 | 0.0058 | 3.21E-09 | 0.000628238 | 35.38072847 | chronic obstructive pulmonary disease | no |
| rs56104760 | 1 | 22486029 | ALFA | G | A | 0.1905 | -0.0747 | 0.0074 | 7.38E-24 | 0.001807204 | 101.8972191 | bone density | no |
| rs633995 | 1 | 172186729 | DNM3 | A | G | 0.4251 | 0.0351 | 0.0058 | 1.61E-09 | 0.000650266 | 36.62206366 | bone density | no |
| rs7548588 | 1 | 110475971 | CSF1 | C | T | 0.391 | 0.0367 | 0.0058 | 2.21E-10 | 0.000710857 | 40.03692448 | bone density | no |
| rs10048745 | 2 | 68962137 | ARHGAP25 | A | G | 0.2477 | -0.0389 | 0.0067 | 6.44E-09 | 0.000598556 | 33.70809155 | neutrophil count,platelet count | no |
| rs10490046 | 2 | 40630678 | SLC8A1 | C | A | 0.2391 | -0.0429 | 0.0067 | 1.43E-10 | 0.000727887 | 40.99676103 | bone density | no |
| rs10931982 | 2 | 202832130 | FZD7 | C | T | 0.7903 | 0.0508 | 0.009 | 1.59E-08 | 0.000565733 | 31.85862098 | bone density | no |
| rs11898505 | 2 | 54684557 | SPTBN1 | G | A | 0.6674 | -0.0342 | 0.006 | 1.28E-08 | 0.000576918 | 32.4888455 | bone density | no |
| rs11904127 | 2 | 85484818 | TCF7L1 | A | G | 0.5512 | -0.0324 | 0.0057 | 1.18E-08 | 0.000573728 | 32.30910119 | bone density | no |
| rs12612325 | 2 | 119632252 | EN1 | A | G | 0.2122 | -0.0548 | 0.0078 | 1.98E-12 | 0.000876206 | 49.35787787 | bone density | no |
| rs144279715 | 2 | 119548256 | LINC01956 | G | A | 0.0147 | 0.2295 | 0.0294 | 6.18E-15 | 0.001081473 | 60.9333824 | bone density | no |
| rs2289410 | 2 | 42284110 | PKDCC | T | A | 0.132 | -0.0494 | 0.0088 | 2.00E-08 | 0.000559578 | 31.51179344 | body height | no |
| rs2350085 | 2 | 202799604 | FZD7 | C | T | 0.1274 | 0.0643 | 0.0085 | 3.79E-14 | 0.001015682 | 57.22274166 | bone density | no |
| rs7586085 | 2 | 166577489 | CSRNP3 | G | A | 0.4663 | -0.0532 | 0.0057 | 8.64E-21 | 0.001545315 | 87.1080157 | bone density | no |
| rs838721 | 2 | 234303405 | DGKD | G | A | 0.563 | 0.0314 | 0.0057 | 4.48E-08 | 0.000538878 | 30.34548984 | bone density | no |
| rs74394007 | 3 | 156692207 | LEKR1 | C | A | 0.138 | -0.0608 | 0.0083 | 2.46E-13 | 0.000952472 | 53.65813098 | bone density | no |
| rs11934731 | 4 | 88831249 | SPP1 | A | G | 0.6738 | -0.0674 | 0.0061 | 8.39E-29 | 0.002164383 | 122.0800478 | bone density | no |
| rs76051363 | 4 | 1006987 | FGFRL1 | T | C | 0.1491 | -0.0794 | 0.0085 | 1.39E-20 | 0.001547909 | 87.25447724 | bone density | no |
| rs11745493 | 5 | 122847622 | HMGB3P17 | G | A | 0.2537 | -0.0445 | 0.0065 | 7.74E-12 | 0.000832045 | 46.86815701 | bone density | no |
| rs7728694 | 5 | 88288341 | MEF2C-AS1 | T | G | 0.461 | -0.0503 | 0.0059 | 1.30E-17 | 0.001289694 | 72.68026704 | bone density | no |
| rs818427 | 5 | 112221869 | SRP19 | T | C | 0.3118 | 0.0342 | 0.0061 | 2.37E-08 | 0.000558168 | 31.43236866 | bone density | no |
| rs13204965 | 6 | 127167072 | RSPO3 | C | A | 0.229 | -0.0619 | 0.007 | 1.02E-18 | 0.001387386 | 78.19334382 | bone density | no |
| rs6557155 | 6 | 151910126 | CCDC170 | G | T | 0.5682 | 0.0751 | 0.0059 | 2.56E-37 | 0.002870401 | 162.0169373 | bone density | no |
| rs7740042 | 6 | 151971720 | ESR1 | A | T | 0.2024 | -0.0494 | 0.0071 | 2.71E-12 | 0.000859367 | 48.40851585 | bone density | no |
| rs7741085 | 6 | 44636919 | SUPT3H | T | C | 0.5874 | 0.0423 | 0.0057 | 1.51E-13 | 0.00097751 | 55.07006523 | bone density | no |
| rs12534510 | 7 | 120730944 | CPED1 | C | A | 0.5545 | 0.0395 | 0.0057 | 3.15E-12 | 0.00085249 | 48.02076202 | body height | no |
| rs1548607 | 7 | 50901491 | GRB10 | G | A | 0.313 | -0.0363 | 0.0066 | 4.18E-08 | 0.000537164 | 30.24892509 | bone density | no |
| rs34102936 | 7 | 38142840 | SFRP4 | A | G | 0.5897 | 0.0471 | 0.0057 | 1.87E-16 | 0.00121166 | 68.27735213 | bone density | no |
| rs34670419 | 7 | 99130834 | ZKSCAN5 | T | G | 0.0394 | -0.088 | 0.0154 | 1.09E-08 | 0.000579812 | 32.65190093 | hormone measurement, cortisol:DHEAS ratio measurement | no |
| rs3801387 | 7 | 120974765 | WNT16 | G | A | 0.2721 | 0.1347 | 0.0063 | 1.15E-100 | 0.008056678 | 457.1288805 | bone density | no |
| rs6465511 | 7 | 96134115 | SEM1 | G | C | 0.6752 | 0.0738 | 0.006 | 1.03E-34 | 0.002680769 | 151.284624 | bone density | no |
| rs6960249 | 7 | 96660132 | DLX5 | G | T | 0.4091 | -0.0325 | 0.0057 | 1.45E-08 | 0.000577273 | 32.50884786 | bone density | no |
| rs73169678 | 7 | 150953205 | SMARCD3 | A | C | 0.1117 | 0.0619 | 0.0091 | 1.05E-11 | 0.000821404 | 46.26825078 | erythrocyte count | no |
| rs73305797 | 7 | 30997087 | ALFA | T | A | 0.2431 | -0.0422 | 0.0067 | 2.40E-10 | 0.000704343 | 39.66978658 | bone density | no |
| rs73719807 | 7 | 121191251 | CYCSP19 | C | A | 0.0871 | 0.0925 | 0.0112 | 1.14E-16 | 0.001210421 | 68.20747737 | bone density | no |
| rs757138 | 7 | 27989403 | JAZF1 | G | T | 0.3111 | 0.0348 | 0.0063 | 3.33E-08 | 0.000541823 | 30.51138742 | prostate carcinoma | no |
| rs10901216 | 9 | 133471891 | FUBP3 | A | G | 0.3438 | -0.0474 | 0.0061 | 5.53E-15 | 0.001071634 | 60.3783973 | body height | no |
| rs10788264 | 10 | 124015986 | BTBD16 | A | G | 0.4815 | -0.0338 | 0.0057 | 2.61E-09 | 0.000624349 | 35.16156985 | bone density | no |
| rs1159798 | 10 | 54412493 | LNCAROD | C | A | 0.7598 | -0.0429 | 0.007 | 1.01E-09 | 0.000666874 | 37.55805312 | blood protein measurement | no |
| rs12258451 | 10 | 54423853 | LNCAROD | G | C | 0.1307 | -0.0702 | 0.0089 | 2.41E-15 | 0.001104154 | 62.21266111 | bone density | no |
| rs73349318 | 10 | 112245400 | DUSP5-DT | T | A | 0.1262 | 0.0472 | 0.0085 | 2.68E-08 | 0.000547549 | 30.83406001 | bone density | no |
| rs10832520 | 11 | 15816918 | ALFA | A | T | 0.0394 | 0.1123 | 0.0158 | 1.00E-12 | 0.000896749 | 50.51611068 | bone density | no |
| rs11228240 | 11 | 68218290 | LRP5 | T | C | 0.2574 | -0.083 | 0.0067 | 1.72E-35 | 0.002719187 | 153.45857 | bone density | no |
| rs143187557 | 11 | 47284279 | NR1H3 | T | C | 0.0223 | -0.1237 | 0.0203 | 1.15E-09 | 0.000659289 | 37.13059348 | bone density | no |
| rs2553773 | 11 | 35083633 | PDHX | G | C | 0.5863 | 0.037 | 0.0058 | 1.49E-10 | 0.000722518 | 40.6941544 | uterine fibroid | no |
| rs35199438 | 11 | 16630779 | SOX6 | T | G | 0.3035 | -0.0489 | 0.0062 | 2.36E-15 | 0.001104001 | 62.20408508 | reticulocyte count | no |
| rs4757350 | 11 | 15703674 | LINC02751 | T | C | 0.7852 | -0.0564 | 0.0069 | 3.75E-16 | 0.001185659 | 66.81048031 | Hair color, High light scatter reticulocyte percentage of red cells | no |
| rs55781332 | 11 | 242859 | PSMD13 | G | A | 0.2169 | 0.0552 | 0.0069 | 8.07E-16 | 0.001135799 | 63.99772582 | mean platelet volume | no |
| rs61884327 | 11 | 46766890 | CKAP5 | C | T | 0.0978 | 0.0801 | 0.0099 | 4.63E-16 | 0.001161729 | 65.46048376 | bone density | no |
| rs634277 | 11 | 86887931 | TMEM135 | G | A | 0.3322 | -0.0607 | 0.0061 | 2.15E-23 | 0.001756181 | 99.01529361 | bone density | no |
| rs7105860 | 11 | 27306364 | CCDC34 | C | G | 0.6025 | -0.0468 | 0.0059 | 2.36E-15 | 0.001116651 | 62.91761482 | bone density | no |
| rs725670 | 11 | 121913230 | MIR100HG | A | G | 0.383 | -0.0322 | 0.0059 | 3.61E-08 | 0.000528924 | 29.78463536 | bone density | no |
| rs1037011 | 12 | 107302778 | EEF1B2P4 | C | T | 0.5208 | 0.0404 | 0.0057 | 1.54E-12 | 0.000891745 | 50.23397977 | bone density | no |
| rs10735851 | 12 | 53743064 | SP7 | A | G | 0.7083 | -0.0541 | 0.0063 | 5.84E-18 | 0.001308458 | 73.73912821 | bone density | no |
| rs10777212 | 12 | 90334829 | LINC02399 | T | G | 0.3455 | 0.0452 | 0.006 | 5.05E-14 | 0.001007284 | 56.74909451 | bone density | no |
| rs117557198 | 12 | 49655948 | TUBA1C | G | A | 0.0676 | 0.0769 | 0.012 | 1.58E-10 | 0.000729102 | 41.06527684 | bone density | no |
| rs118115924 | 12 | 49379537 | WNT1 | T | G | 0.0139 | -0.2822 | 0.0301 | 6.99E-21 | 0.001559259 | 87.89528833 | body height | no |
| rs35125553 | 12 | 1639249 | WNT5B | G | A | 0.2855 | 0.0383 | 0.0066 | 5.20E-09 | 0.00059795 | 33.67396408 | bone density | no |
| rs78667121 | 13 | 43200103 | TNFSF11 | A | G | 0.0325 | 0.1326 | 0.018 | 1.70E-13 | 0.000963249 | 54.26584942 | alkaline phosphatase measurement | no |
| rs9594738 | 13 | 42952145 | LINC02341 | T | C | 0.4592 | -0.0614 | 0.0057 | 3.84E-27 | 0.002057348 | 116.030349 | blood protein measurement | no |
| rs1286150 | 14 | 91464890 | RPS6KA5 | C | T | 0.1955 | 0.0549 | 0.0072 | 2.44E-14 | 0.001031921 | 58.13855903 | bone density | no |
| rs12442242 | 15 | 38340874 | LINC02345 | G | A | 0.1499 | 0.0509 | 0.0082 | 4.94E-10 | 0.00068411 | 38.52941609 | bone density | no |
| rs2414098 | 15 | 51537806 | MIR4713HG | C | T | 0.6102 | 0.0329 | 0.0059 | 1.99E-08 | 0.000552157 | 31.09369542 | endometrial endometrioid carcinoma | no |
| rs3743347 | 15 | 67547301 | IQCH | A | C | 0.2351 | 0.0519 | 0.0068 | 1.75E-14 | 0.00103391 | 58.25074146 | risk-taking behaviour | no |
| rs71390846 | 16 | 86714715 | FOXL1 | C | G | 0.1836 | -0.0484 | 0.0075 | 1.38E-10 | 0.00073937 | 41.64403128 | bone density | no |
| rs8047501 | 16 | 392318 | AXIN1 | G | A | 0.5077 | -0.0524 | 0.0059 | 1.13E-18 | 0.001399476 | 78.87568032 | bone density | no |
| rs144691710 | 17 | 41819562 | SOST | G | A | 0.0751 | 0.1017 | 0.0113 | 2.24E-19 | 0.001437062 | 80.99712174 | bone density | no |
| rs2873195 | 17 | 2064702 | SMG6 | T | A | 0.6873 | 0.0406 | 0.0062 | 4.31E-11 | 0.000761295 | 42.87984982 | high density lipoprotein cholesterol measurement | yes |
| rs8070128 | 17 | 17804725 | TOM1L2 | T | C | 0.5763 | -0.0394 | 0.0059 | 1.98E-11 | 0.000791698 | 44.5936466 | bone density | no |
| rs9910055 | 17 | 42283037 | UBTF | T | C | 0.2624 | 0.0442 | 0.0067 | 3.12E-11 | 0.000772635 | 43.51905946 | appendicular lean mass | no |
| rs9972944 | 17 | 63771079 | CEP112 | G | A | 0.5951 | -0.0363 | 0.0059 | 6.87E-10 | 0.000672098 | 37.85243255 | bone density | no |
| rs884205 | 18 | 60054857 | TNFRSF11A | C | A | 0.7579 | 0.0531 | 0.0068 | 4.39E-15 | 0.001082221 | 60.97555813 | alkaline phosphatase measurement | no |
| rs6029130 | 20 | 39103882 | LINC01370 | T | C | 0.2874 | 0.0348 | 0.0063 | 3.50E-08 | 0.000541823 | 30.51138742 | bone density | no |
| rs6040063 | 20 | 10640877 | JAG1 | G | A | 0.4993 | -0.0359 | 0.0056 | 1.78E-10 | 0.000729644 | 41.0957973 | bone density | no |
| rs11910328 | 21 | 40350744 | LINC01700 | A | G | 0.8351 | -0.0429 | 0.0077 | 2.99E-08 | 0.000551199 | 31.03971332 | bone density | no |
| rs1452102 | 21 | 28773868 | RPL10P1 | G | T | 0.4129 | 0.0345 | 0.0057 | 1.74E-09 | 0.000650461 | 36.63304726 | bone density | no |
| rs9976876 | 21 | 36970350 | RUNX1 | T | G | 0.447 | -0.0375 | 0.0058 | 8.01E-11 | 0.000742163 | 41.80142777 | bone density | no |

**Supplementary Table 2.**

Characteristics of the overall stroke or its subtypes associated genetic instrumental variables included in the MR study,and the reported traits of selected genetic instrumental variables of overall stroke or its subtypes searched in GWAS Catalog.

| SNP | chr | pos | Gene | EA | OA | eaf | Beta | Se | P | R2 | F | Trait | Excluded from MR analysis |
| --- | --- | --- | --- | --- | --- | --- | --- | --- | --- | --- | --- | --- | --- |
| **stroke** |  |  |  |  |  |  |  |  |  |  |  |  |  |
| rs1052053 | 1 | 156202173 | PMF1 | G | A | 0.3984 | -0.0624 | 0.0082 | 2.70E-14 | 0.00012962 | 57.90812859 | stroke | no |
| rs10776752 | 1 | 113044328 | WNT2B | T | G | 0.1583 | 0.0715 | 0.0128 | 2.50E-08 | 6.98E-05 | 31.20255805 | pulse pressure measurement | no |
| rs11191833 | 10 | 105619678 | STN1 | A | G | 0.3942 | -0.0527 | 0.0082 | 1.66E-10 | 9.25E-05 | 41.30394951 | cortical surface area measurement | no |
| rs11957829 | 5 | 121515195 | ZNF474 | G | A | 0.175 | -0.0654 | 0.0116 | 1.62E-08 | 7.12E-05 | 31.78612403 | stroke | no |
| rs12445022 | 16 | 87575332 | JPH3 | A | G | 0.3089 | 0.0574 | 0.0089 | 1.05E-10 | 9.31E-05 | 41.59506689 | non-lobar intracerebral hemorrhage | no |
| rs16896398 | 6 | 43262704 | TTBK1 | T | A | 0.3383 | 0.0477 | 0.0084 | 1.30E-08 | 7.22E-05 | 32.24602909 | hypertension | no |
| rs2107595 | 7 | 19049388 | TWIST1 | A | G | 0.2204 | 0.0727 | 0.0096 | 4.86E-14 | 0.000128369 | 57.34881007 | coronary artery disease,peripheral arterial disease | no |
| rs2129977 | 4 | 111712432 | PITX2 | G | A | 0.6835 | -0.073 | 0.0091 | 9.40E-16 | 0.000144042 | 64.35184326 | atrial fibrillation | no |
| rs35436 | 12 | 115554523 | UBA52P7 | T | C | 0.3834 | -0.0462 | 0.0083 | 2.86E-08 | 6.94E-05 | 30.983168 | Diuretic use measurement,systolic blood pressure | no |
| rs4959130 | 6 | 1356916 | LINC01394 | A | G | 0.1381 | 0.0779 | 0.0129 | 1.42E-09 | 8.16E-05 | 36.46645532 | stroke | no |
| rs6825454 | 4 | 155501188 | FGA | C | T | 0.3036 | 0.0518 | 0.0087 | 2.61E-09 | 7.94E-05 | 35.45023102 | venous thromboembolism | no |
| rs7859727 | 9 | 22102165 | CDKN2B-AS1 | T | C | 0.5299 | 0.0494 | 0.0079 | 4.22E-10 | 8.75E-05 | 39.1018919 | healthspan | no |
| rs8103309 | 19 | 11174935 | SMARCA4 | C | T | 0.345 | -0.0501 | 0.0091 | 3.40E-08 | 6.79E-05 | 30.31033404 | stroke | no |
| rs880315 | 1 | 10796866 | CASZ1 | C | T | 0.4 | 0.0527 | 0.0084 | 3.62E-10 | 8.81E-05 | 39.36050971 | pulse pressure measurement,triglyceride measurement | no |
| rs9316222 | 13 | 47214690 | LRCH1 | C | T | 0.7794 | 0.0599 | 0.0096 | 4.31E-10 | 8.71E-05 | 38.93222586 | Platelet distribution width | no |
| **ischemic_stroke** |  |  |  |  |  |  |  |  |  |  |  |  | no |
| rs11242678 | 6 | 1337180 | FGF1 | T | C | 0.255 | 0.0723 | 0.0114 | 2.70E-10 | 9.13E-05 | 40.22211648 | blood lipid levels | no |
| rs2066864 | 4 | 155525695 | FGG | A | G | 0.2452 | 0.0634 | 0.0115 | 3.51E-08 | 6.90E-05 | 30.39351034 | factor VIII measurement, venous thromboembolism | no |
| rs2107595 | 7 | 19049388 | TWIST1 | A | G | 0.1673 | 0.0882 | 0.0132 | 2.33E-11 | 0.000101384 | 44.64649143 | coronary artery disease,peripheral arterial disease | no |
| rs2634074 | 4 | 111677041 | PITX2 | A | T | 0.7877 | -0.0941 | 0.0121 | 5.90E-15 | 0.000137332 | 60.47926904 | pulse pressure measurement, sex interaction measurement | no |
| rs2758612 | 1 | 156205301 | LOC1 | C | T | 0.3547 | -0.0653 | 0.0111 | 3.68E-09 | 7.86E-05 | 34.60815382 | diabetes | no |
| rs34311906 | 4 | 113732090 | ANK2 | C | T | 0.4024 | 0.0649 | 0.0113 | 1.07E-08 | 7.49E-05 | 32.98606679 | stroke | no |
| rs473238 | 11 | 102700360 | WTAPP1 | C | T | 0.8674 | -0.0831 | 0.0147 | 1.65E-08 | 7.26E-05 | 31.95695606 | FEV/FVC ratio, response to bronchodilator | no |
| **large_artery_atherosclerosis_IS** |  |  |  |  |  |  |  |  |  |  |  |  | no |
| rs12124533 | 1 | 115657799 | NGF-AS1 | T | C | 0.2412 | 0.1529 | 0.0268 | 1.22E-08 | 7.93E-05 | 32.54942094 | stroke | no |
| rs17612742 | 4 | 148414651 | EDNRA | C | T | 0.209 | 0.176 | 0.0261 | 1.46E-11 | 0.000110764 | 45.47180616 | peripheral arterial disease | no |
| rs2107595 | 7 | 19049388 | TWIST1 | A | G | 0.2375 | 0.1871 | 0.0238 | 3.65E-15 | 0.000150533 | 61.80043683 | coronary artery disease,peripheral arterial disease | no |
| rs72985562 | 11 | 102800278 | MMP13 | G | T | 0.0834 | 0.2449 | 0.0439 | 2.42E-08 | 7.58E-05 | 31.12048909 | FEV/FVC ratio, response to bronchodilator | no |
| **small_vessel_IS** |  |  |  |  |  |  |  |  |  |  |  |  | no |
| rs12445022 | 16 | 87575332 | JPH3 | A | G | 0.2764 | 0.1198 | 0.0201 | 2.55E-09 | 0.000179338 | 35.52361344 | stroke | no |
| **cardioembolic_IS** |  |  |  |  |  |  |  |  |  |  |  |  | no |
| rs12932445 | 16 | 73069888 | ZFHX3 | C | T | 0.2077 | 0.1839 | 0.0213 | 6.86E-18 | 0.000180325 | 74.54219037 | stroke | no |
| rs13143308 | 4 | 111714419 | PITX2 | G | T | 0.7183 | -0.2798 | 0.0193 | 1.86E-47 | 0.000508265 | 210.1738601 | stroke | no |
| rs1906613 | 4 | 111769663 | ALFA | A | G | 0.3723 | 0.1019 | 0.0181 | 1.83E-08 | 7.67E-05 | 31.69488036 | Arrhythmia | no |
| rs55884259 | 5 | 172642370 | BNIP1 | A | G | 0.6576 | -0.1096 | 0.0186 | 3.87E-09 | 8.40E-05 | 34.72107143 | QRS duration | no |
| **intracerebral_hemorrhage** |  |  |  |  |  |  |  |  |  |  |  |  | no |
| rs116161367 | 1 | 58210169 | DAB1 | A | C | 0.0403809 | 0.4516 | 0.0804 | 1.98E-08 | 6.66E-05 | 31.54960561 | intracerebral hemorrhage | no |
| rs12946564 | 17 | 1963961 | HIC1 | A | G | 0.784517 | -0.1899 | 0.0338 | 1.85E-08 | 6.67E-05 | 31.56564693 | cortical thickness | no |
